# Naturally acquired antibody response to a *Plasmodium falciparum* chimeric vaccine candidate GMZ2.6c and its components (MSP-3, GLURP, and Pfs48/45) in individuals living in Brazilian malaria-endemic areas

**DOI:** 10.1186/s12936-021-04020-6

**Published:** 2022-01-04

**Authors:** Barbara Oliveira Baptista, Ana Beatriz Lopes de Souza, Evelyn Kety Pratt Riccio, Cesare Bianco-Junior, Paulo Renato Rivas Totino, João Hermínio Martins da Silva, Michael Theisen, Susheel Kumar Singh, Linda Eva Amoah, Marcelo Ribeiro-Alves, Rodrigo Medeiros Souza, Josué Costa Lima-Junior, Cláudio Tadeu Daniel-Ribeiro, Lilian Rose Pratt-Riccio

**Affiliations:** 1 Laboratório de Pesquisa em Malária, Instituto Oswaldo Cruz (IOC), Fundação Oswaldo Cruz (Fiocruz), Rio de Janeiro, Brazil; 2 Centro de Pesquisa, Diagnóstico e Treinamento em Malária, Fiocruz, Secretaria de Vigilância em Saúde, Ministério da Saúde, Brazil; 3 Departamento de Bioinformática, Fiocruz, Ceará, Brazil; 4 Centre for Medical Parasitology at Department of International Health, Immunology and Microbiology, University of Copenhagen and Department of Infectious Diseases, Copenhagen University Hospital, Rigshospitalet, Copenhagen, Denmark; 5 Immunology Department, Noguchi Memorial Institute for Medical Research, University of Ghana, Accra, Ghana; 6 Laboratório de Pesquisa Clínica em DST e AIDS, Instituto Nacional de Infectologia Evandro Chagas, Fiocruz, Rio de Janeiro, Brazil; 7 Laboratório de Doenças Infecciosas na Amazônia Ocidental, Universidade Federal do Acre, Acre, Brazil; 8 Laboratório de Imunoparasitologia, IOC, Rio de Janeiro, Fiocruz Brazil

**Keywords:** Malaria, *Plasmodium falciparum*, Immune response, Antibodies, GMZ2.6c, Vaccine

## Abstract

**Background:**

The GMZ2.6c malaria vaccine candidate is a multi-stage *Plasmodium falciparum* chimeric protein which contains a fragment of the sexual-stage Pfs48/45-6C protein genetically fused to GMZ2, a fusion protein of GLURP and MSP-3, that has been shown to be well tolerated, safe and immunogenic in clinical trials performed in a malaria-endemic area of Africa. However, there is no data available on the antigenicity or immunogenicity of GMZ2.6c in humans. Considering that circulating parasites can be genetically distinct in different malaria-endemic areas and that host genetic factors can influence the immune response to vaccine antigens, it is important to verify the antigenicity, immunogenicity and the possibility of associated protection in individuals living in malaria-endemic areas with different epidemiological scenarios. Herein, the profile of antibody response against GMZ2.6c and its components (MSP-3, GLURP and Pfs48/45) in residents of the Brazilian Amazon naturally exposed to malaria, in areas with different levels of transmission, was evaluated.

**Methods:**

This study was performed using serum samples from 352 individuals from Cruzeiro do Sul and Mâncio Lima, in the state of Acre, and Guajará, in the state of Amazonas. Specific IgG, IgM, IgA and IgE antibodies and IgG subclasses were detected by Enzyme-Linked Immunosorbent Assay.

**Results:**

The results showed that GMZ2.6c protein was widely recognized by naturally acquired antibodies from individuals of the Brazilian endemic areas with different levels of transmission. The higher prevalence of individuals with antibodies against GMZ2.6c when compared to its individual components may suggest an additive effect of GLURP, MSP-3, and Pfs48/45 when inserted in a same construct. Furthermore, naturally malaria-exposed individuals predominantly had IgG1 and IgG3 cytophilic anti-GMZ2.6c antibodies, an important fact considering that the acquisition of anti-malaria protective immunity results from a delicate balance between cytophilic/non-cytophilic antibodies. Interestingly, anti-GMZ2.6c antibodies seem to increase with exposure to malaria infection and may contribute to parasite immunity.

**Conclusions:**

The data showed that GMZ2.6c protein is widely recognized by naturally acquired antibodies from individuals living in malaria-endemic areas in Brazil and that these may contribute to parasite immunity. These data highlight the importance of GMZ2.6c as a candidate for an anti-malarial vaccine.

**Supplementary Information:**

The online version contains supplementary material available at 10.1186/s12936-021-04020-6.

## Background

Malaria, an infectious parasitic disease caused by blood-borne apicomplexan parasites of the genus *Plasmodium*, remains a tremendous burden to global public health, causing 405,000 deaths annually and generating morbidity to more than two hundred million of individuals [1]. Among the seven *Plasmodium* species able to infect humans, *Plasmodium falciparum* is responsible for most of the severe cases and deaths [1]. Control, elimination, and the ultimate eradication of malaria will require effective therapeutics. Artemisinin-based combination therapy (ACT) is currently the first-line of therapy for uncomplicated *P. falciparum* malaria infection in most parts of the world; however, resistance to artemisinin and to its partner combination drugs has developed in the Greater Mekong Subregion (GMS) and more recently in South America [2–7], highlighting the need for the development and implementation of an effective vaccine [8]. Malaria vaccine efforts have focused on determining which of the antigens expressed by *P. falciparum* are targets of protective immunity.

**Fig. 1:**
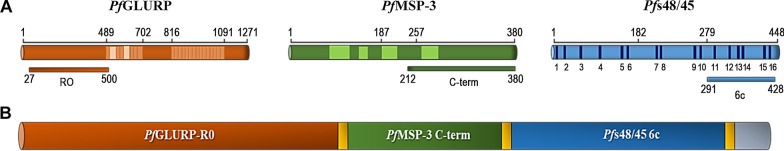
Schematic representation of *Pf*GLURP, *Pf*MSP3, *Pf*s48/45 and the GMZ2.6c hybrid protein. A. Schematic representation of *Pf*GLURP (red), showing N-terminal R0 Region; *Pf*MSP-3 (green), showing C-terminal Regions and *Pf*s48/45 (blue), showing the 6c Region. B. Schematic representation of GMZ2.6C fusion protein ( Adapted from 11 and 12)

The GMZ2.6c malaria vaccine candidate is a multi-stage *P. falciparum* chimeric protein that contains a fragment of the sexual-stage Pfs48/45-6C protein genetically fused to recombinant protein GMZ2, an asexual-stage vaccine construct consisting of the N-terminal region of the Glutamate-Rich Protein (GLURP) and the C-terminal region of Merozoite Surface Protein-3 (MSP-3) [9, 10] (Fig. 1).

GLURP is expressed in both pre-erythrocytic and erythrocytic stages in the vertebrate host and may participate in the binding to receptors on erythrocytes surface during merozoite invasion and formation of the parasitophorous vacuole; MSP-3 is expressed in the erythrocytic stage and has a crucial role not only in binding to the host red blood cell, but also in protecting the parasite against haem that is released during parasite egression; Pfs48/45 is expressed on the surface of gametocytes and gametes playing an important role in fertilization, thus considered a candidate for the transmission-blocking vaccine [11–15]. GLURP, MSP-3 and Pfs48/45 vaccine candidates have been selected based on their immunogenicity in natural conditions of exposure, correlation between specific antibody titers with degrees of clinical protection of individuals living in endemic areas and/or the ability of antibodies to inhibit the *P. falciparum* in vitro growth [16–26]. A recent study with GMZ2.6c showed that this chimera elicits functional antibodies in mice and that the formulations containing synthetic TLR4 agonist glucopyranosyl lipid adjuvant (*GLA*) or synthetic lipid adjuvant (*SLA*) induce high parasite-specific antibody titers, IFN-γ responses in CD4 + TH1 cells, and a high percentage of multifunctional CD4 + T cells expressing IFN-γ and TNF in response to GMZ2.6c [9].

Until now, there is no data available on the antigenicity or immunogenicity of GMZ2.6c in humans. However, clinical trials performed in a malaria-endemic area of Africa showed that GMZ2, the vaccine construct of GLURP and MSP-3, was well tolerated, safe, and immunogenic [27–31]. The immunization with GMZ2 elicited high levels of specific and cytophilic IgG antibodies which mediated parasite killing in vitro as measured by antibody-dependent cellular inhibition (ADCI) assays, and decreased the incidence of clinical malaria in individuals with higher concentration of vaccine-induced anti-GMZ2 IgG antibodies [27–31]. Moreover, high antibody titers naturally acquired against GMZ2 and its components have been observed in individuals living in highly endemic areas in Africa [28, 30, 32] and are associated with lower parasite densities [33]. It is also believed that GLURP and MSP-3 has a synergistic effect, since higher prevalence individuals with antibodies as higher titers of antibodies were detected against GMZ2 compared to either of its components separately, in naturally exposed populations [32].

Considering that circulating parasites can be genetically distinct in different malaria-endemic areas and host genetic factors can influence the immune response to vaccine antigens, it is important to verify the antigenicity, immunogenicity, and the possibility of associated protection in malaria-endemic areas with different epidemiological scenarios. The goal of this work is, therefore, to evaluate the profile of antibody response against the chimeric protein GMZ2.6c and its components (MSP-3, GLURP and Pfs48/45) in residents of the Brazilian Amazon, naturally exposed to malaria, in areas with different levels of malaria transmission.

## Methods

### Study area and volunteers

**Fig. 2:**
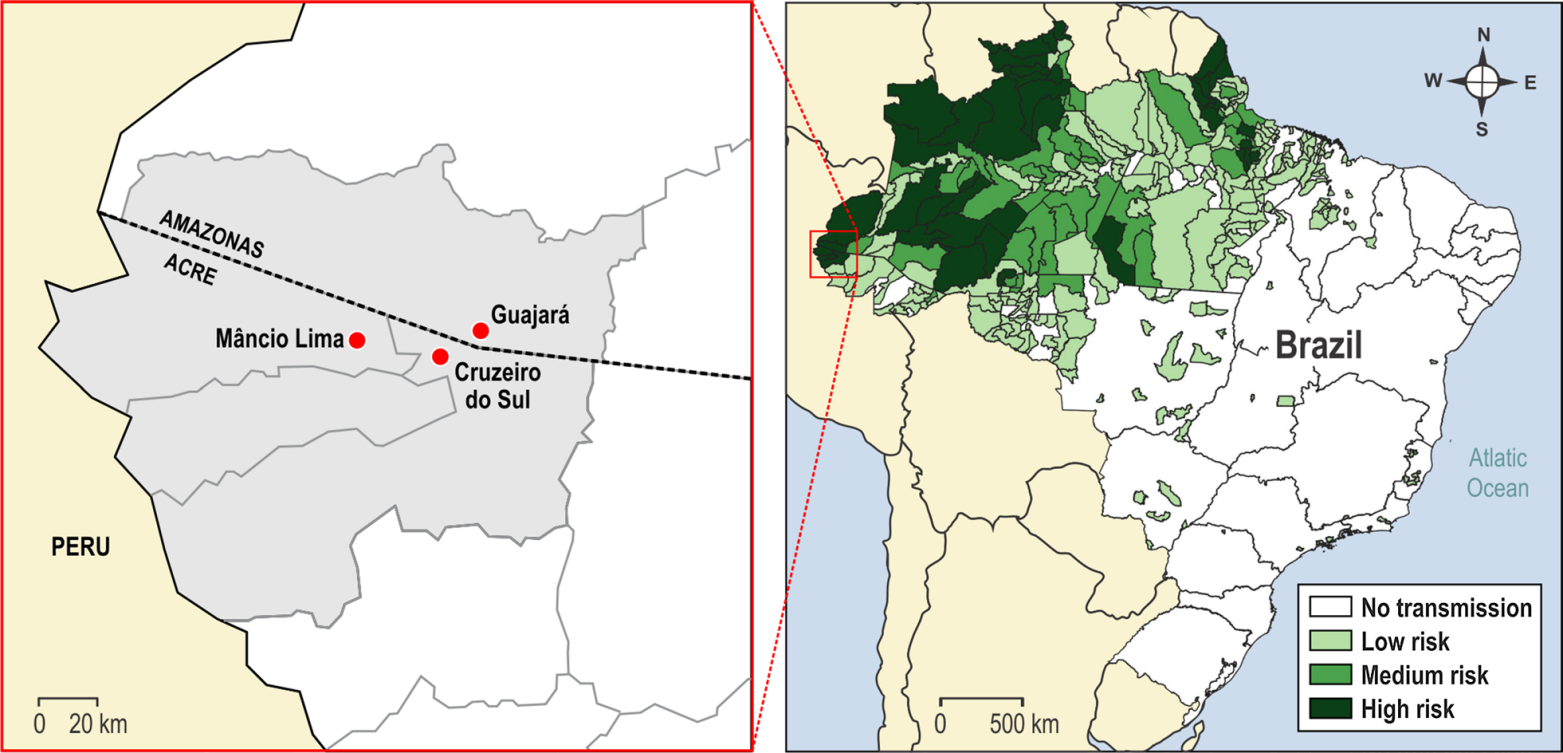
Map of Brazil showing the malaria-endemic areas with their respective transmission levels. In detail, the studied areas of Cruzeiro do Sul (CZS) and Mâncio Lima (ML), state of Acre, and Guajará (GJ) state of Amazonas

A cross-sectional cohort study was carried out from June to August 2016, in three malaria-endemic areas of the Brazilian Amazon, where 99% of autochthonous cases are reported. Two of them, Cruzeiro do Sul (07º37′50’’S/72º40′13’’W) and Mâncio Lima (07º36′49’’S/72º53′47’’W), are high-risk areas situated at the Juruá Valley, state of Acre, and correspond, respectively, to the 3^rd^ and 6^th^ main hotspots, among the 17 municipalities that concentrate 80% of falciparum malaria transmission in the whole Amazon. The Guajará municipality (02º58′18’’S/57º40′38’W), a medium-risk area in the state of Amazonas (Fig. 2), was also included. [34]. In Brazil, the risk of contracting malaria is measured by the Annual Parasitological Indexes (API), which serves to classify transmission areas into high (≥ 50), medium (< 50 and ≥ 10), low risk (< 10 and > 1) and very low risk (< 1), according to the number of autochthonous cases per 1,000 inhabitants. In 2016, a year of sample collection, Cruzeiro do Sul, Mâncio Lima and Guajará registered, respectively, 5447, 1432 and 674 cases of falciparum malaria, and the API of 85.3, 67.3 and 43.3 for *P. falciparum*.

Serum samples were obtained from 299 malaria exposed individuals from Cruzeiro do Sul (CZS group, n = 124), Mâncio Lima (ML group, n = 88) and Guajará (GJ group, n = 87). In addition, serum samples from individuals who have never had malaria were included in the study as area control (CO group, n = 53). All individuals from this group were negative for the presence of malaria parasites as assessed by thick blood films. Non-endemic control blood samples from 40 individuals from the laboratory staff (Rio de Janeiro, Brazil) who had neither history of malaria nor contact with malaria transmission areas, were included as Rio de Janeiro Controls.

### Ethics statement

The study protocol was reviewed and approved by the Oswaldo Cruz Foundation Ethical Committee (CEP-FIOCRUZ CAAE 46,084,015.1.0000.5248), which included obtaining the following patients’ written consents for research use of their blood samples. Written informed consent was obtained from all adult donors or from donor’s parents in the cases of children. All the procedures adopted in this study fully complied with specific federal permits issued by the Brazilian Ministry of Health.

### Epidemiological survey, malaria diagnosis, and blood sampling

Donors who provided informed consent also completed an epidemiological survey. In order to evaluate the degree of malaria exposure, subjects responded to questions related to personal data such as: age, time of residence in the endemic area, number of previous malaria episodes, the time elapsed from the last infection, use of malaria prophylaxis, and presence of symptoms.

Venous peripheral blood (20 ml) was collected into heparin or EDTA tubes for antibodies analyses and molecular diagnosis, respectively. The plasma was stored at –20 °C, and the pellets, containing peripheral blood cells collected into EDTA tubes, were mixed with equal volumes of a cryopreservation solution (0.9% NaCl/4.2% sorbitol/20% glycerol) and were stored at –70 °C for later DNA extraction for molecular diagnosis. Thin and thick blood smears were examined for identification of malaria parasites by a technician experienced in malaria diagnosis from the Laboratory of Malaria Research (Fiocruz), which is the headquarters of the CEMART (Center for Malaria Research and Training), a reference center for malaria diagnosis in the Extra-Amazonian Region for the Brazilian Ministry of Health. Malaria diagnosis was performed in Giemsa-stained thin and thick blood smears, and parasitological evaluation was done by examination of 200 fields at 1,000 × magnification under oil-immersion. Thin blood smears of the positive samples were examined for species identification. The parasitaemia was expressed as the number of parasites/μl of blood in the thick blood smear. The number of parasites/μl of blood was calculated by multiplying the number of parasites counted against 500 leucocytes, and the number of leukocytes of the subject and dividing the product by 500. To increase the sensitivity of the parasite detection, molecular analyses were performed in all samples. Briefly, DNA was extracted from the blood samples using the QIAamp DNA blood midi kit (Qiagen, Germantown, MD, USA) according to manufacturer instructions, and Polymerase Chain Reaction (PCR) was performed using specific primers for genus (*Plasmodium* sp) and species (*P. falciparum* and *Plasmodium vivax*), as previously described [35]. Positive donors for *P*. *vivax* and/or *P*. *falciparum* at the time of blood collection were subsequently treated by the chemotherapeutic regimen recommended by the Brazilian Ministry of Health [36].

### Recombinant proteins and antibody assays

The multi-stage GMZ2.6c construct was created from GLURP_79-1500_ and MSP-3_462–747_ fragments genetically fused to the Pfs48/45_859–1284_ region (6c). GMZ2.6c and its fragments were expressed in *Lactococcus lactis* strain MG1363 and purified as previously described [9]. Briefly, *L. lactis* containing pSS4 was cultured in LAB medium supplemented 5 mM cysteamine/0.5 mM cystamine. The recombinant protein in the supernatant of the culture was purified by affinity chromatography with a 5 ml HisTrap™ HP column (GE Healthcare, Sweden) followed by a 5 ml HiTrap NHS-activated HP column containing monoclonal antibody mAb45.1 (epitope I), according to the manufacturer (GE Healthcare, Sweden). To assess purity in purified proteins, reversed-phase HPLC was performed, showing a relative purify ˃ 95%. Production of GLURP-R0, MSP3 and Pfs48/45-6C was done as previously described [37, 38].

Microtiter 96-well plates (Maxisorp, NUNC, Denmark) were coated with the recombinant proteins at an optimal dilution using phosphate-buffered saline at pH 7.2 (PBS) or a carbonate-bicarbonate buffer at pH 9.6 at 100 µl/well overnight at 4 °C (Additional file 1: Table S1). The plates were washed, the uncoated sites were blocked and then plasma samples diluted 1:100 in dilution buffer were added in duplicate wells for each individual. The plates were washed, 100 µl of peroxidase-conjugated mouse anti-human IgG, IgM, IgA, or IgE (Sigma, St. Louis, MO), 1:1,000 in dilution buffer were added, and the plates were incubated for 1 h at RT or 37 °C. To detect specific IgG subclass, plates were incubated with peroxidase-conjugated mouse anti-human IgG1, IgG2, IgG3, or IgG4 (clones 4E3 for IgG1, 31–7-4 for IgG2, HP6050 for IgG3 and HP6025 for IgG4; SouthernBiotech, Birmingham, AL, USA) 1:1,000 in dilution buffer for 1 h at RT or 37 °C. After washing, 100 µl of a solution of 0.4 mg/ml orthophenylenediamine (OPD, Sigma) and H2O2 30% (Merck) in citrate–phosphate buffer at pH 5.0 (24 mM citric acid, Sigma, and 51 mM dibasic sodium phosphate, Sigma) were added to each well, the plates were incubated for 5 min at room temperature in the dark, and then 50 µl/well of 2 N H2SO4 (Merck) were added. Optical density (OD) was identified at 492 nm using a SpectraMax 250 ELISA reader (Molecular Devices, Sunnyvale, CA, USA). Sera from five Rio de Janeiro controls were used to establish the normal range for each assay. The cut-off values were determined as the OD mean plus 3 standard deviations (SD) of the Rio de Janeiro controls. The results were expressed as a semi-quantitative Reactivity Indices (RIs), which were calculated by the OD mean of each tested individual divided by cut-off value. Subjects were scored as responders if the RI against each recombinant protein was higher than 1.0.

### Statistical analyses

Data were stored in the Epi-Info 6 (*Centers for Disease Control and Prevention, Atlanta, GA*) data bank software. Epi-Info and GraphPad Instat (*GraphPad Software, Inc*) statistical software programs were used for data analyses. Kruskal–Wallis followed by Mann–Whitney pairwise tests were used to analyse the differences in distributions of continuous numerical variables, and the chi-square analysis was applied to test independence of prevalence among groups. The Spearman rank coefficient test was used to analyse the correlation between variables.

## Results

### Population characteristics

The main characteristics of the studied groups are shown in Table 1. CZS, ML, and GJ groups were not different in terms of gender, age, natural exposure to malaria infection, the time elapsed since the last malaria episode, and *P. falciparum* or *P. vivax* parasitaemia. However, the group ML showed a higher number of past malaria episodes than CZS and GJ, as well as GJ group had a longer time since symptom onset (number of past malaria episodes: P = 0.01, ML versus CZS; P = 0.0008, ML versus GJ; time of symptoms: P = 0.003, GJ versus CZS and ML).

**Table 1:** Personal, clinical, and epidemiological characteristics of the studied populations

		CZS *n* = 124	ML *n* = 88	GJ *n* = 87	CO n = 53
*Personal Data*					
Gender	Male	66 (53.2%)	44 (50%)	25 (51.7%)	16 (30.2%)
	Female	58 (46.8%)	44 (50%)	42 (48.3%)	37 (69.8%)*
Age (years)		33 ± 15.2	35.3 ± 15.8	36.9 ± 17	22.8 ± 6.2**
Time of residence in malaria-endemic area (years)		32.8 ± 15	34.3 ± 16.6	36.9 ± 17	21.7 ± 7.7**
*Clinical and Epidemiological Data*					
Number of past malaria episodes		10.2 ± 13	13 ± 13.3***	8.9 ± 10.3	NA
Time elapsed since the last malaria episode (months)		60 ± 97	28 ± 45	48 ± 73	NA
Time of symptoms (days)		4 ± 4	6 ± 14	8 ± 8 ****	NA
Diagnosis	*P. falciparum* *P. vivax*	25 (20.2%) 39 (31.4%)	12 (13.6%) 25 (28.4%)	6 (6.7%) 11 (12.8%)	NA NA
Parasitaemia (parasites/μl of blood)	*P. falciparum* *P. vivax*	16,000 ± 20,092 19,142 ± 17,559	5600 ± 3577 23,294 ± 15,507	8000 ± 5656 10,666 ± 8326	NA NA

^*^P = 0.008, CO *versus* CZS; *P* = 0.03, CO *versus* ML; *P* = 0.02 CO *versus* GJ. **P < 0.0001, CO *versus* CZS, ML and GJ. *** *P* = 0.01, ML *versus* CZS; *P* = 0.0008, ML *versus* GJ. ****P = 0.003, GJ *versus* CZS; *P* = 0.003, GJ *versus* ML. *n*: number of individuals. Age, time of residence in malaria-endemic areas, number of previous malaria episodes, time elapsed since the last malaria episodes, time of symptoms and parasitaemia represent median ± IQR (interquartile range) and were compared by Kruskal–Wallis followed by Mann–Whitney pairwise tests. Prevalence of gender and infective *Plasmodium* species at the time of collection (diagnosis) inter groups was compared by Chi-squared tests. NA: Not analysed

All individuals of the CO group were residents of the studied areas, CZS, ML or GJ but reported no previous malaria episode, and none had symptoms or circulating parasites at the time of collection. CO group showed a higher frequency of females and a lower mean of age and time of residence in malaria endemic area than CZS, ML and GJ groups (gender: P = 0.008, CO versus CZS; P = 0.03, CO versus ML; P = 0.02 CO versus GJ; age and time of residence in malaria endemic area: P < 0.0001, CO versus CZS, ML and GJ, for all analyses).

### Prevalence of antibody response against GMZ2.6c and its components, GLURP, MSP-3, and Pfs48/45

**Fig. 3:**
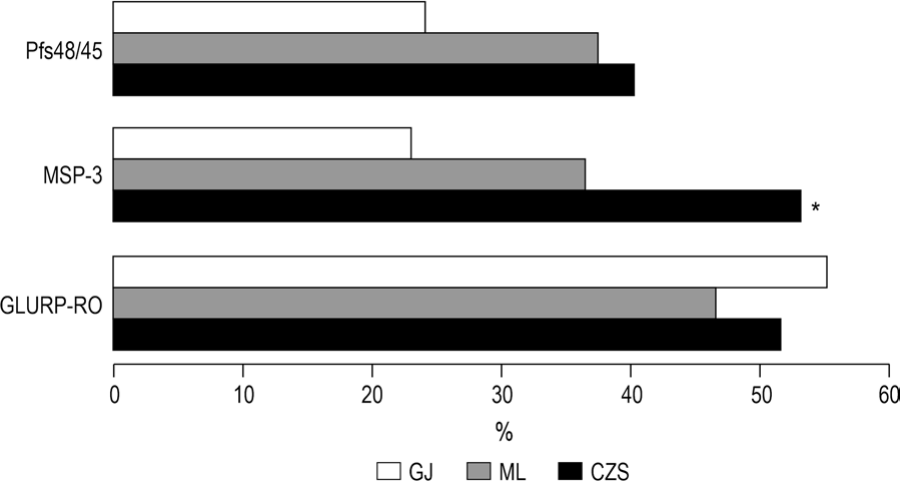
Prevalence of individuals presenting antibodies that recognizes GLURP, MSP-3, and Pfs48/45. The data refer to the prevalence of individuals with IgG, IgM, IgA and/or IgE detectable antibodies in each group studied. *P < 0.01, CZS MSP-3 *versus* ML MSP-3; *P* < 0.0001, CZS MSP-3 *versus* GJ MSP-3. Prevalence of responders were compared by Chi-squared tests

Considering the prevalence of individuals with detectable antibodies that recognize GMZ2.6c, regardless antibody class (whether IgG, IgM, IgA and/or IgE), GMZ2.6c was shown to be largely recognized by naturally acquired antibodies in the CZS, ML and GJ groups, and these three groups showed comparable prevalence of individuals presenting GMZ2.6c specific antibodies: 103/124 (83%), 65/88 (74%), and 63/87 (72%), respectively. The analyses of the prevalence of individuals with detectable antibodies that recognize GMZ2.6c components, regardless antibody class (whether IgG, IgM, IgA and/or IgE), is shown in Fig. 3. The prevalence of individuals with antibodies against GLURP and Pfs48/45 was similar in CZS, ML and GJ groups. However, the CZS group presented a higher prevalence of anti-MSP-3 positive individuals than the ML and GJ groups (P < 0.01, CZS versus ML; P < 0.0001, CZS versus GJ).

No volunteers from the CO group, who reported no previous malaria episodes, had antibodies against GMZ2.6c, GLURP, MSP-3, or Pfs48/45. Likewise, none of the 40 Rio-controls, with no history of malaria or previous travel to malaria transmission areas, had detectable antibodies to GMZ2.6c, GLURP, MSP-3, or Pfs48/45.

**Fig. 4:**
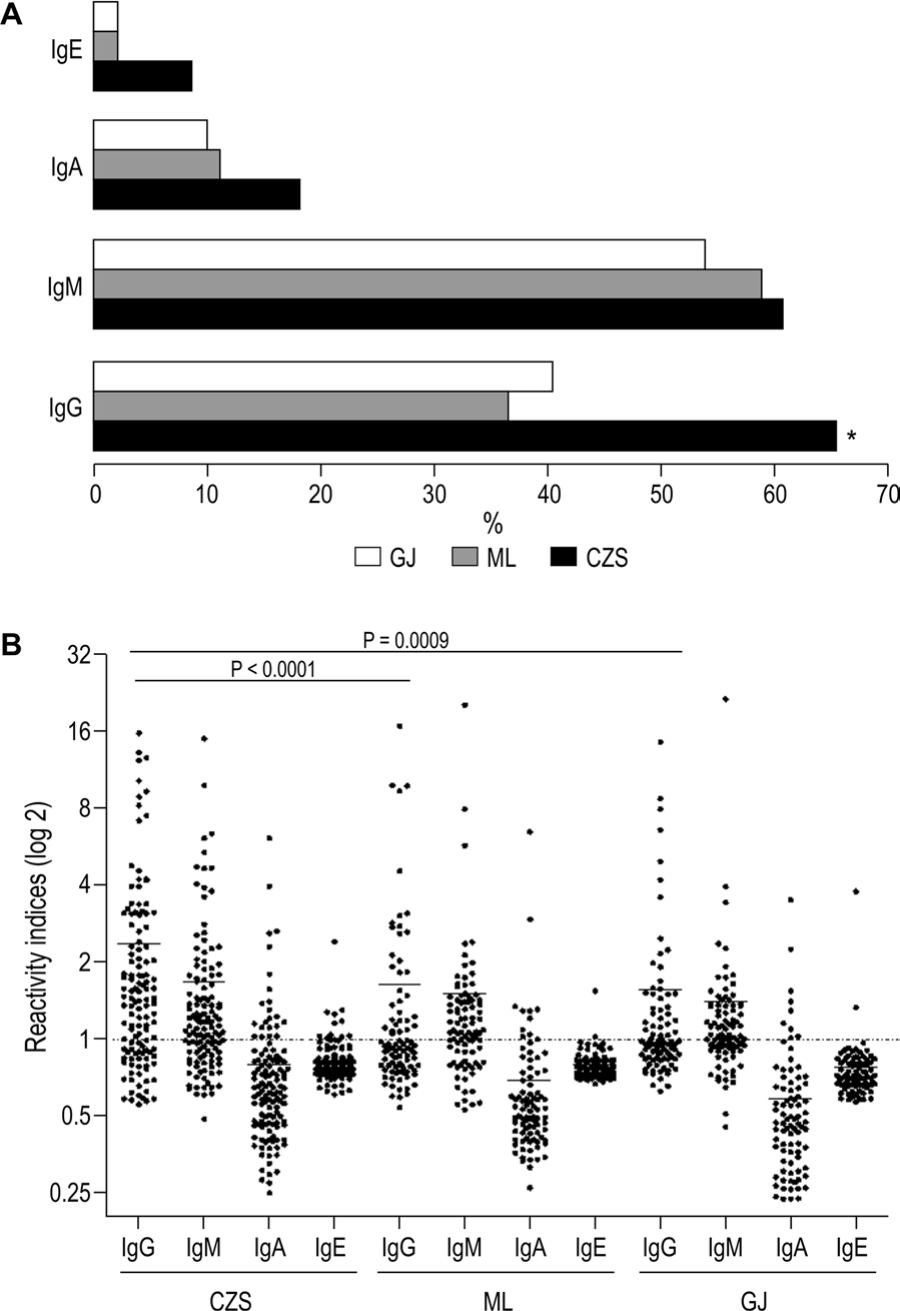
Prevalence (A) and Reactivity indices (B) of individuals presenting antibodies that recognize GMZ2.6c. * P < 0.0001, CZS *versus* ML and GJ groups. Reactivity indices are individual values. Dashed line represents the cutoff for the Reactivity Index value that was considered to classify individuals as positive (> 1) and negative (< 1). Lines represent the medians. Prevalence of responders within and between groups were compared by Chi-squared tests and Reactivity Indices were compared by Kruskal–Wallis followed by Mann–Whitney pairwise tests

The prevalence of individuals with IgG, IgM, IgA, and IgE anti-GMZ2.6c antibodies in CZS, ML, and GJ groups is shown in Fig. 4a. A higher frequency of IgG antibody positive individuals was observed in CZS than in ML and GJ groups (P < 0.0001, for both). Not only the frequency but also the levels of anti-GMZ2.6c IgG antibodies were higher in the CZS group (Fig. 4b). Although a high prevalence of individuals with anti-GMZ2.6c IgM antibodies has been observed, no difference was found when comparing the different groups. In contrast, anti-GMZ2.6c IgA and IgE antibodies were uncommon.

**Fig. 5:**
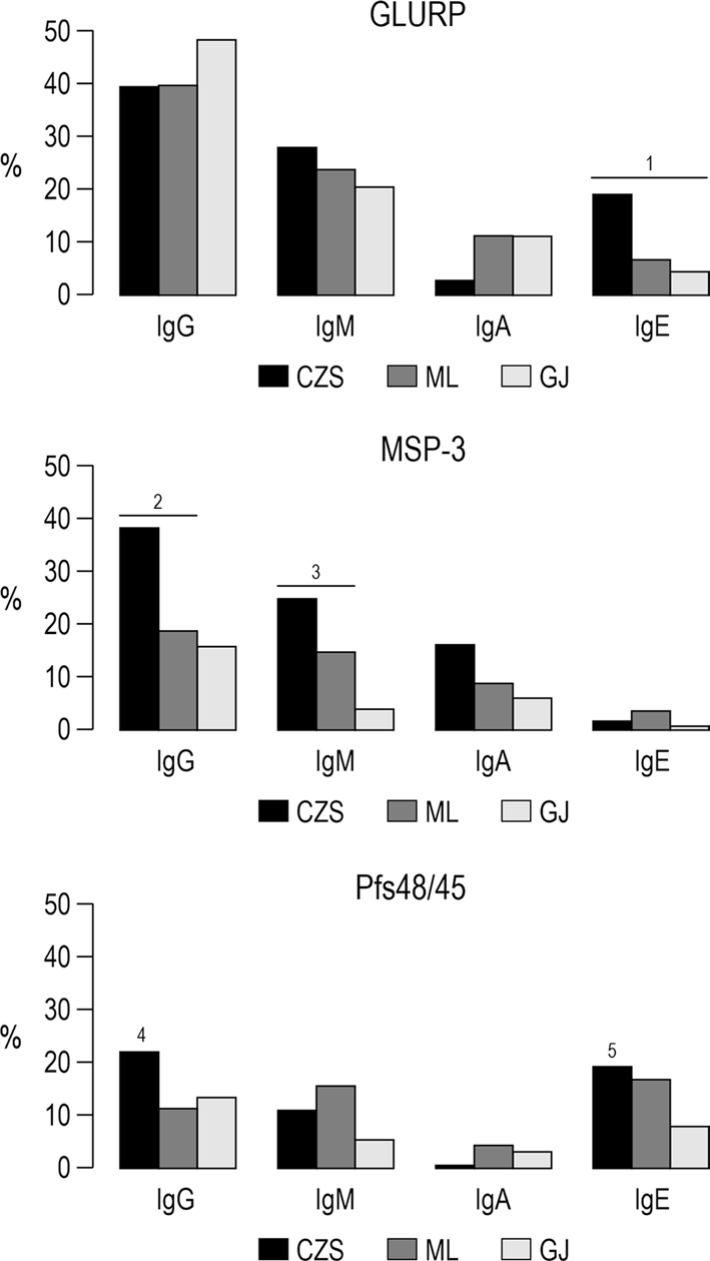
Prevalence of individuals with IgG, IgM, IgA and IgE antibodies against GLURP, MSP-3, and Pfs48/45. ^1^*P* < 0.0001, CZS IgE GLURP versus ML and GJ IgE GLURP. ^2^P = 0.001, CZS IgG MSP-3 versus ML IgG MSP-3, P = 0.0003, CZS IgG MSP-3 versus GJ IgG MSP-3. ^3^P < 0.0001, CZS IgM MSP-3 versus GJ IgM MSP-3. ^4^P = 0.04, CZS IgG Pfs48/45 versus ML IgG Pfs48/45. ^5^P = 0.02, CZS IgE Pfs48/45 versus GJ IgE Pfs48/45. Prevalence of responders within and between groups were compared by Chi-squared tests

**Fig. 6:**
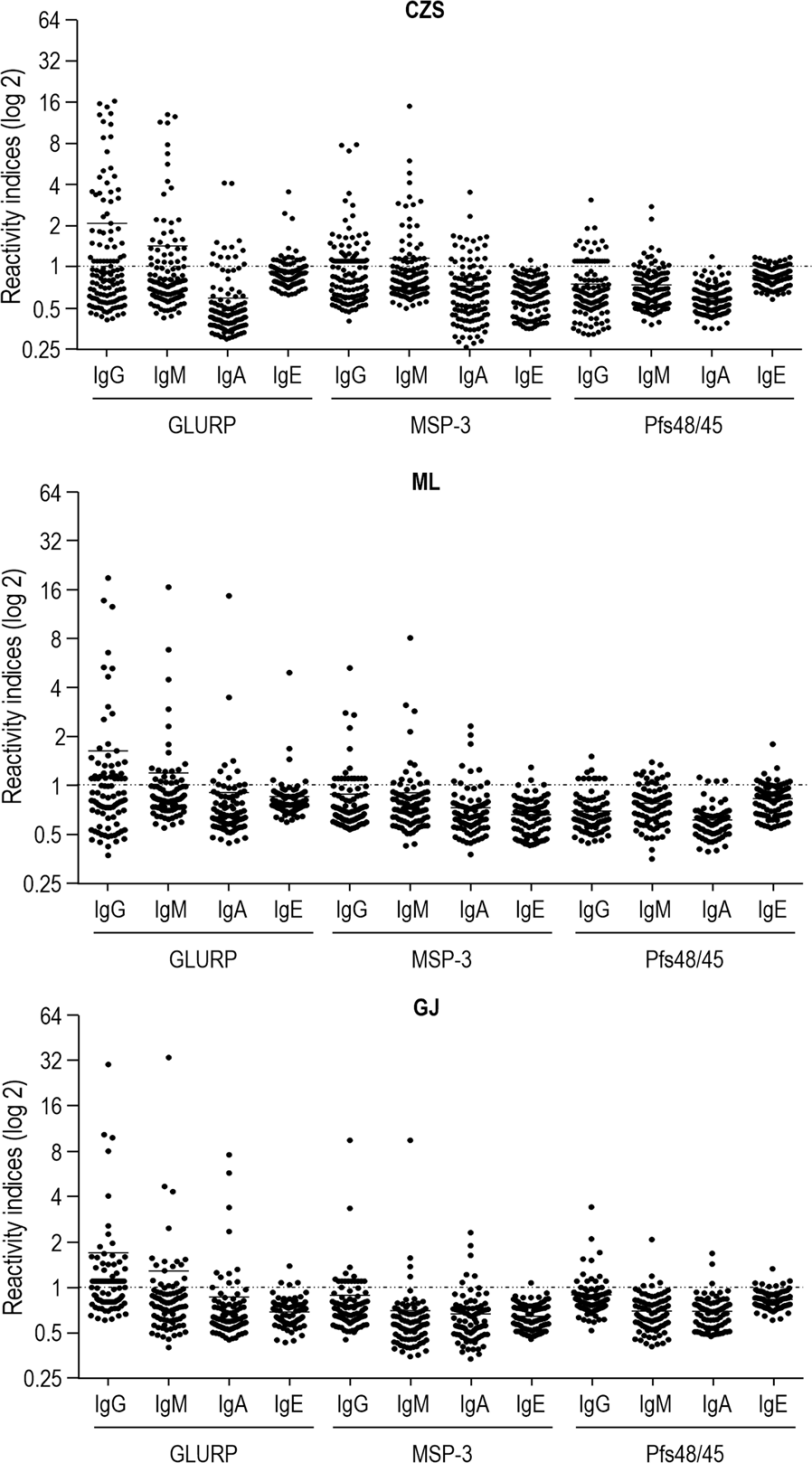
Levels of IgG, IgM, IgA and IgE antibodies (Reactivity indices) against GLURP, MSP-3 and Pfs48/45. Reactivity indices are individual values. Dashed line represents the cutoff for the Reactivity Index value that was considered to classify individuals as positive (> 1) and negative (< 1). Lines represent the medians. Reactivity indices were compared by Kruskal–Wallis followed by Mann–Whitney pairwise tests

As showed in Fig. 5, the prevalence of individuals with IgG, IgM, and IgA antibodies against GLURP were similar in the three studied groups, except for IgE antibodies against GLURP that were more prevalent in CZS than in ML and GJ groups (P < 0.0001, for both). Similarly, the CZS group showed a higher prevalence of individuals with IgG against MSP-3 than ML and GJ groups and IgM antibodies against MSP-3 than GJ group (IgG: P = 0.001, CZS versus ML; P = 0.0003, CZS versus GJ. IgM: P < 0.0001 CZS versus GJ). For Pfs48/45, IgG antibodies were more prevalent in CZS than in the ML group (P = 0.04), while IgE antibodies were more prevalent in CZS than in the GJ group (P = 0.02). The Reactivity Indices of IgG, IgM, IgA and IgE against GLURP, MSP-3 and Pfs48/45 were similar when comparing the CZS, ML and GJ groups (Fig. 6).

### Prevalence of individuals presenting IgG1, IgG2, IgG3, and IgG4 antibodies to GMZ2.6c and to its GLURP, MSP-3, and Pfs48/45 components, in CZS, ML, and GJ groups

**Fig. 7:**
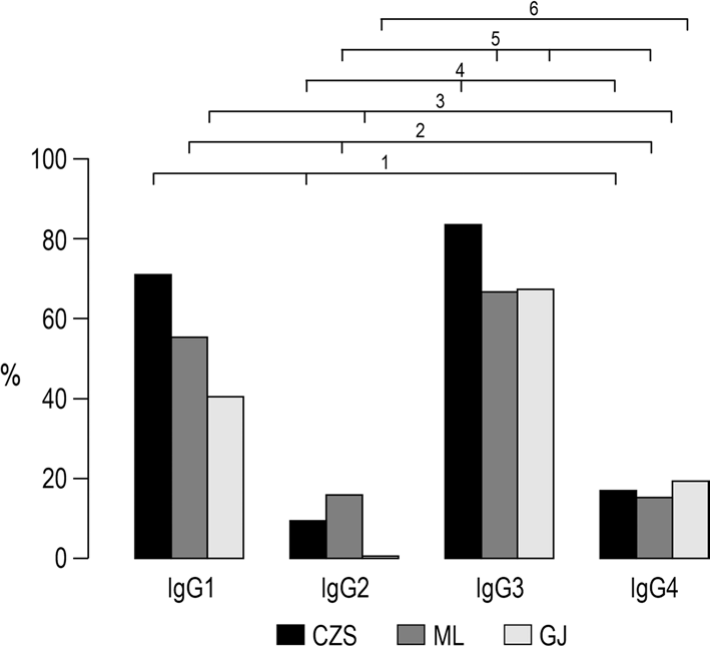
Prevalence of individuals presenting IgG1, IgG2, IgG3 and IgG4 antibodies that recognizes GMZ2.6c. ^1^*P* < 0.0001, CZS IgG1 versus CZS IgG2 and IgG4; ^2^P = 0.01, ML IgG1 versus ML IgG2; P < 0.001, ML IgG1 versus ML IgG4; ^3^P < 0.0001, GJ IgG1 versus GJ IgG2 and IgG4; ^4^P < 0.0001, CZS IgG3 versus CZS IgG2 and IgG4; ^5^P = 0.01, ML IgG3 versus ML IgG2; P < 0.0001ML IgG3 versus ML IgG4; ^6^P < 0.0001; GJ IgG3 versus GJ IgG2 and IgG4. Prevalence of responders were compared by Chi-squared tests

**Fig. 8:**
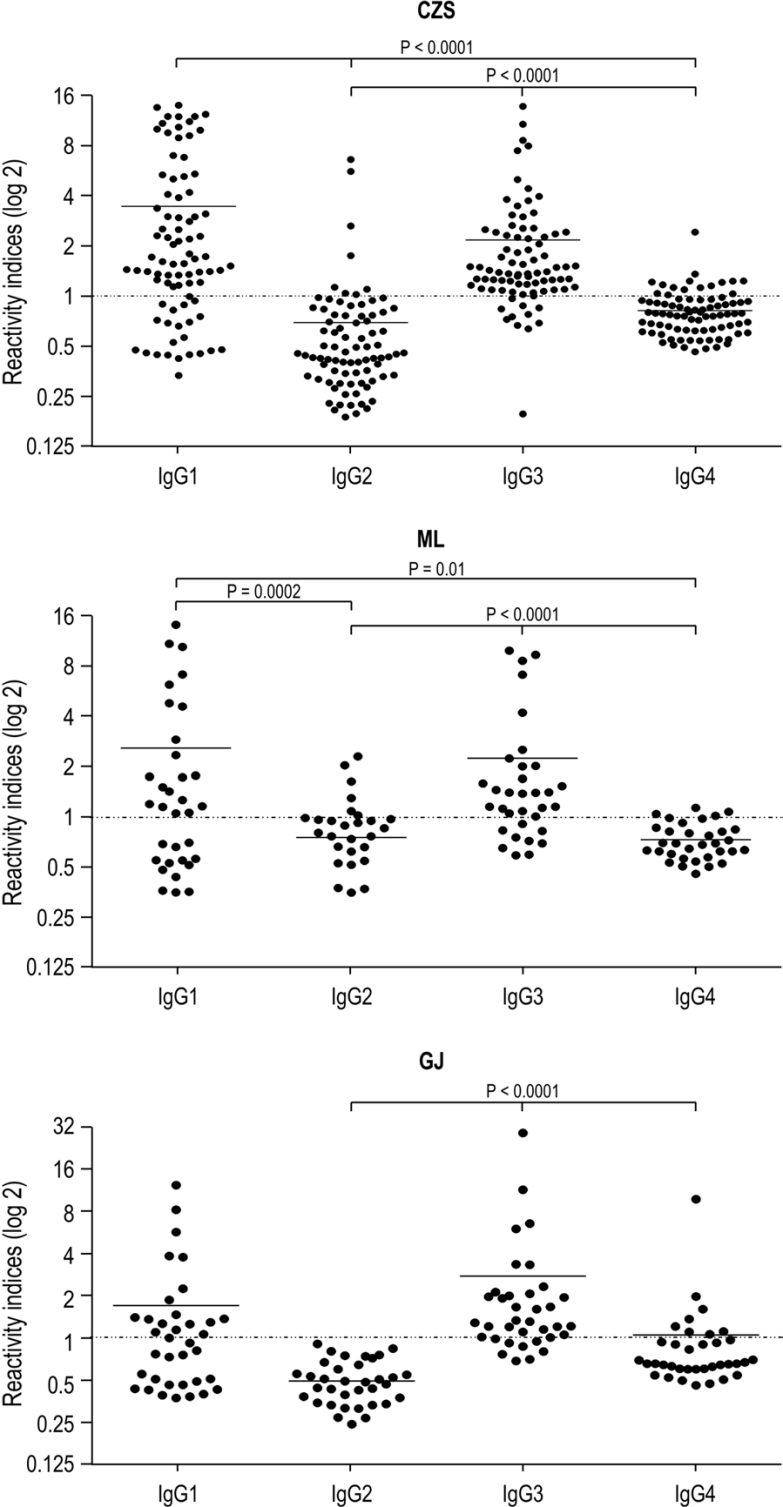
Levels of IgG1, IgG2, IgG3, and IgG4 antibodies (Reactivity indices) against GMZ2.6c. Reactivity indices are individual values. Dashed line represents the cutoff for the Reactivity Index value that was considered to classify individuals as positive (> 1) and negative (< 1). Lines represent the medians. Reactivity indices were compared by Kruskal–Wallis followed by Mann–Whitney pairwise tests

The analyses of the IgG1, IgG2, IgG3 and IgG4 antibodies were performed in all samples with detectable IgG antibodies. Anti-GMZ2.6c antibodies were mainly of cytophilic subclass in all study groups (*P* < 0.0001, CZS and GJ, IgG1 versus IgG2 and IgG4; *P* = 0.01, ML IgG1 versus IgG2; *P* < 0.001, ML IgG1 versus IgG4; *P* < 0.0001, CZS and GJ IgG3 versus IgG2 and IgG4; *P* = 0.01, ML IgG3 *versus* IgG2; *P* < 0.0001ML IgG3 versus IgG4) (Fig. 7). The CZS group showed a higher frequency of individuals with anti-GMZ2.6c IgG1 antibodies than the GJ group (*P* = 0.02), while the frequency of individuals with IgG3 antibodies was not different between the groups. CZS and ML groups also showed higher levels of IgG1 and IgG3 than of IgG2 and IgG4 antibodies (P < 0.0001, CZS IgG1 and IgG3 versus IgG2 and IgG4; P = 0.0002, ML IgG1 versus IgG2, P = 0.01, ML IgG1 versus IgG4; P < 0.0001, ML IgG3 versus IgG2 and IgG4). The GJ group showed higher levels of IgG3 than of IgG2 and IgG4 antibodies (P < 0.0001; for all analyses) (Fig. 8).

**Fig. 9:**
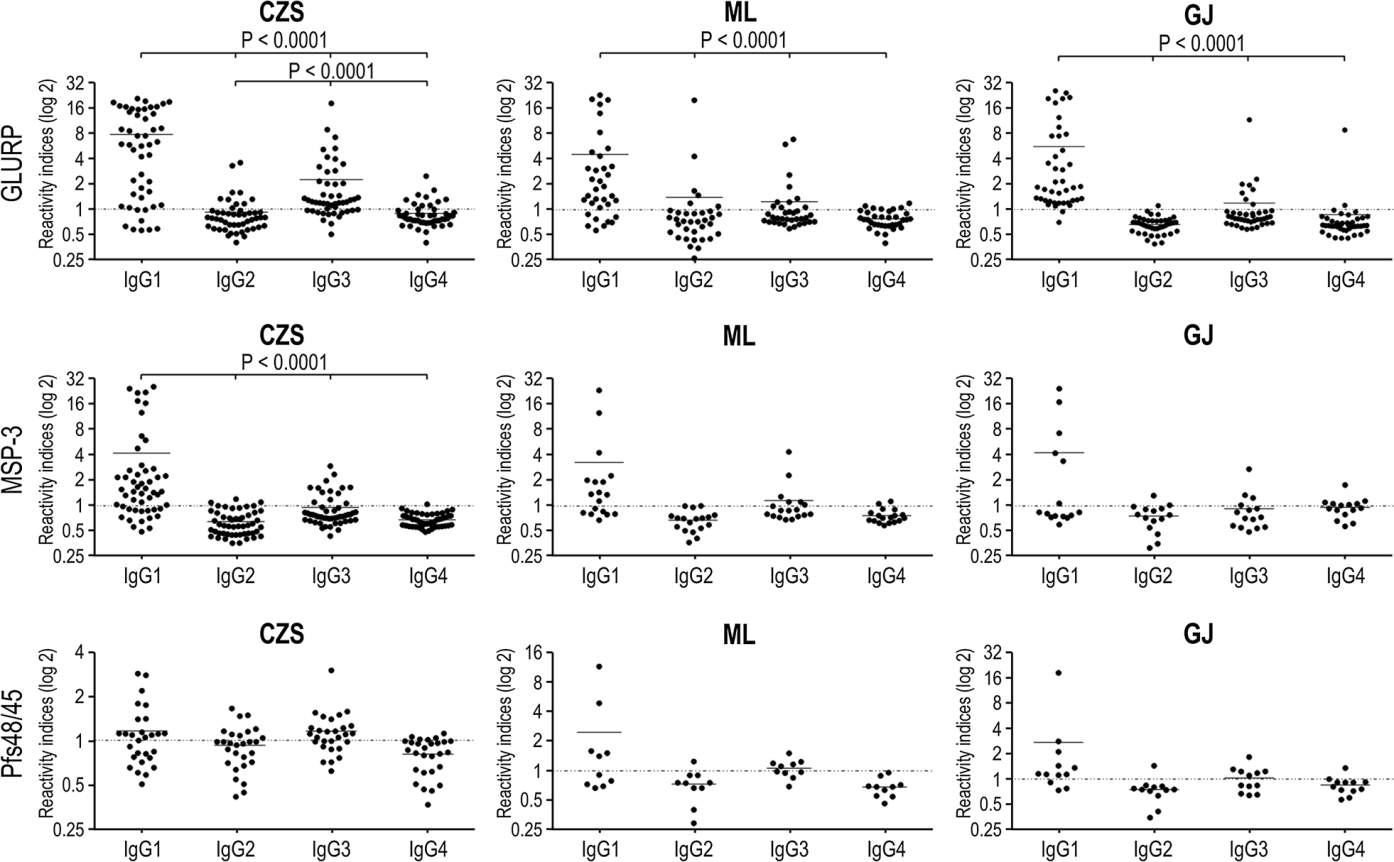
Levels of IgG1, IgG2, IgG3, and IgG4 antibodies (Reactivity Indices) against GLURP, MSP-3, and Pfs48/45. Reactivity indices are individual values. Dashed line represents the cutoff for the Reactivity Index value that was considered to classify individuals as positive (> 1) and negative (< 1). Lines represent the medians. Reactivity indices were compared by Kruskal–Wallis followed by Mann–Whitney pairwise tests

As showed in Table 2, GLURP induced mainly a cytophilic antibody response in CZS group (P < 0.0001, IgG1 and IgG3 versus IgG2 and IgG4) whereas, in ML and GJ groups, GLURP induced mainly an IgG1 antibody response (ML: P < 0.0001, IgG1 versus IgG2, IgG3 and IgG4; GJ: P < 0.0001, IgG1 versus IgG2 and IgG4) as did MSP-3 in the CZS group (P < 0.0001, IgG1 versus IgG2, IgG3, and IgG4). In the three studied groups, the levels of anti-GLURP IgG1 antibodies were higher than the levels of IgG2, IgG3, and IgG4 (P < 0.0001, for all analyses), as well as the levels of IgG3 antibodies that were higher than the levels of IgG2 and IgG4 in CZS group (CZS: P < 0.0001, IgG3 versus IgG2 and IgG4). Also, the levels of anti-MSP-3 IgG1 antibodies were higher than those of IgG2, IgG3, and IgG4 in the CZS group (P < 0.0001, for all analyses) (Fig. 9). No difference was observed in IgG1, IgG2, IgG3, and IgG4 antibody levels between the CZS, ML, and GJ groups for each of the GMZ2.6c components.

**Table 2:** Prevalence of individuals presenting IgG subclass that recognizes GMZ2.6c components in studied groups

Groups	IgG1	IgG2	IgG3	IgG4
GLURP				
CZS	40/46* (87%)	10/46 (21,7%)	33/46* (71,7%)	10/46 (21,7%)
ML	30/34** (88,2%)	7/34 (20,6%)	12/34 (35,3%)	6/34 (21,7%)
GJ	23/24*** (95,8%)	1/24 (4,2%)	9/24 (37,5%)	2/24 (8,3%)
MSP-3				
CZS	28/31**** (90,3%)	0/31 (0%)	8/31 (25,8%)	0/31 (0%)
ML	6/7	0/7	3/7	0/7
GJ	5/5	1/5	4/5	2/5
Pfs48/45				
CZS	2/2	0/2	0/2	1/2
ML	2/2	0/2	0/2	0/2
GJ	3/7	1/7	3/7	4/7

^*^P < 0.0001, IgG1 and IgG3 versus IgG2 and IgG4. **P < 0.0001, IgG1 versus IgG2, IgG3 and IgG4. ***P < 0.0001, IgG1 versus IgG2 and IgG4. ****P < 0.0001, IgG1 versus IgG2, IgG3 and IgG4. Only the IgG positive samples were tested for subclasses. Prevalence of responders within and between Groups were compared by Chi-squared tests

### Antibody levels in *P. falciparum*-infected and non-infected individuals

**Fig. 10:**
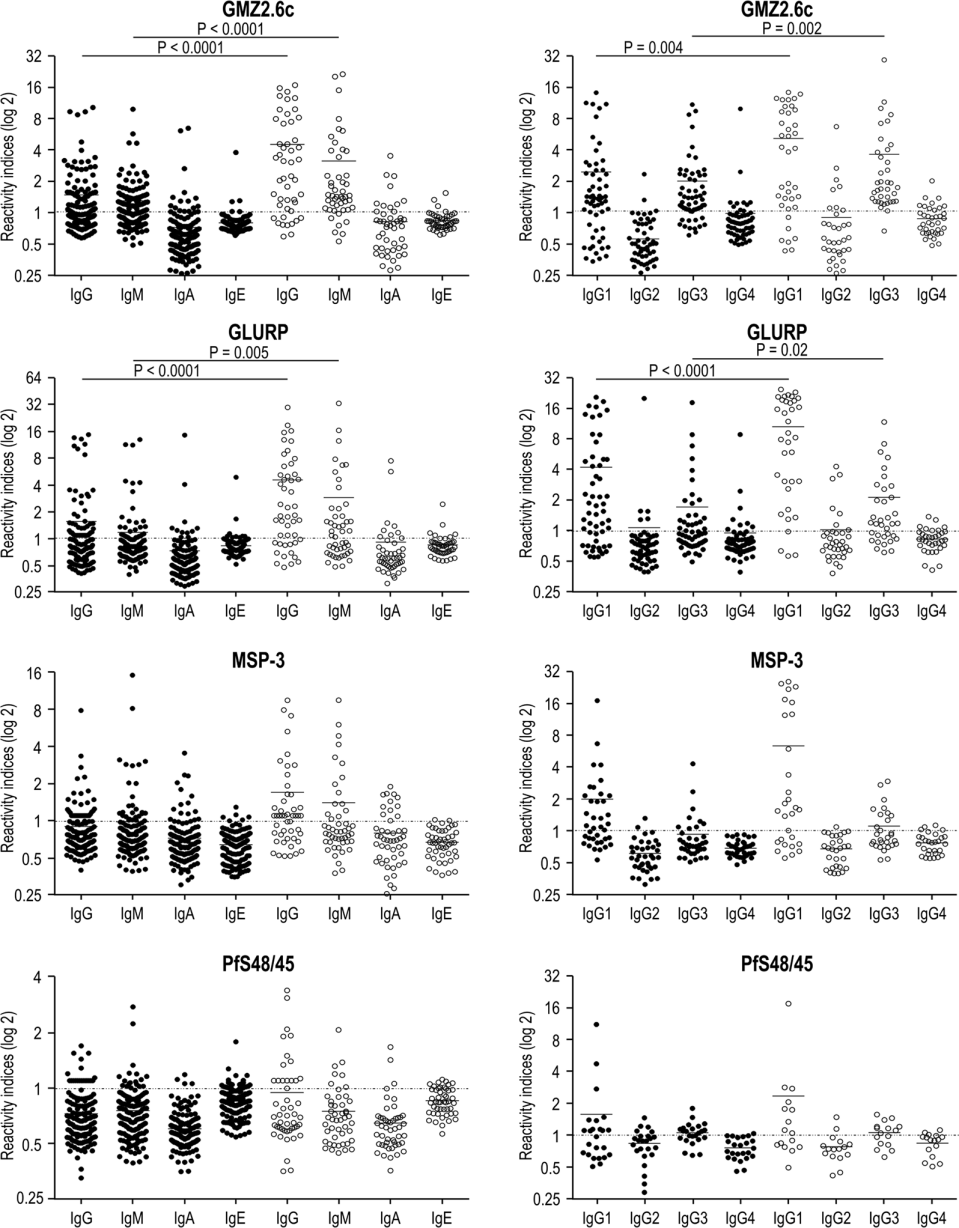
Levels of antibodies against GMZ2.6c and its components in non-infected (•) and infected-individuals (◦). Reactivity indices are individual values. Dashed line represents the cutoff for the Reactivity Index value that was considered to classify individuals as positive (> 1) and negative (< 1). Lines represent the medians. Reactivity indices were compared Kruskal–Wallis followed by Mann Whitney pairwise tests

The levels of IgG, IgM, IgA, IgE, and IgG subclass antibodies to GMZ2.6c and to its components were compared between 43 *P. falciparum*-infected and 181 non-infected individuals, regardless of whether individuals belong to the CZS, ML or GJ group. Levels of anti-GMZ2.6c and anti-GLURP IgG and IgM antibodies were higher in infected than in non-infected individuals (GMZ2.6c: *P* < 0.0001, for both. GLURP: *P* < 0.0001, for IgG and *P* < 0.005 for IgM). Levels of cytophilic (IgG1 and IgG3) antibodies against these antigens were also higher in the *P. falciparum*-infected than in the non-infected (GMZ2.6c: *P* = 0.004 for IgG1; *P* = 0.002, for IgG3. GLURP: P < 0.0001 for IgG1; *P* = 0.02 for IgG3) group. No difference was observed in antibody levels against MSP-3 and Pfs48/45 between infected and non-infected individuals (Fig. 10).

### Associations of antibody responses against GMZ2.6c, GLURP, MSP-3, and Pfs48/45 to personal, clinical and epidemiological data

**Fig. 11:**
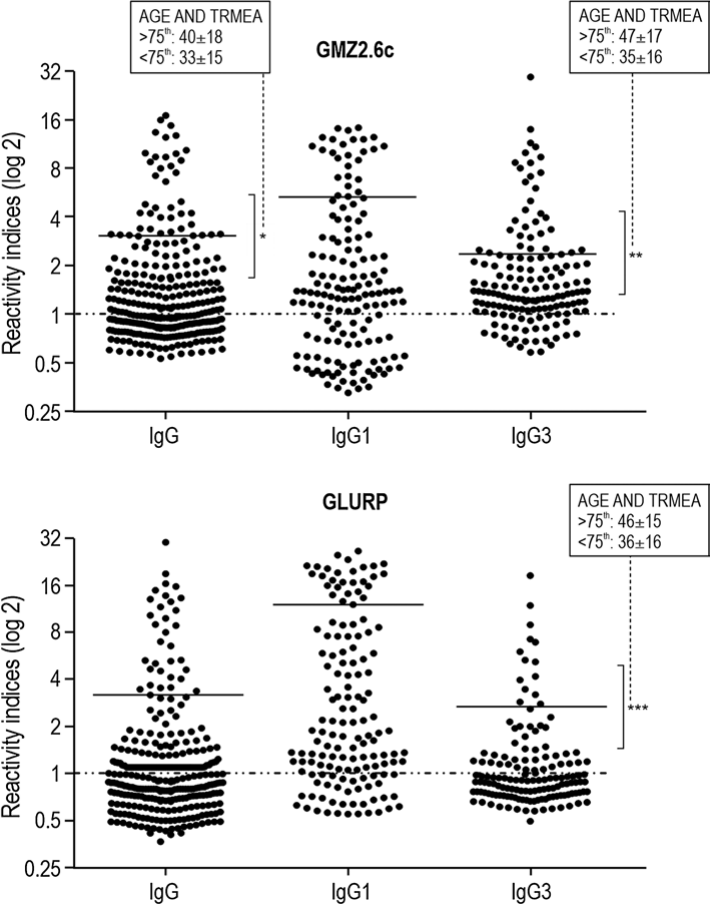
Levels of antibodies (Reactivity Indices) against GMZ2.6c and GLURP according to personal and epidemiological parameters. Reactivity indices are individual values. Dashed line represents the cutoff for the Reactivity Index value that was considered to classify individuals as positive (> 1) and negative (< 1). Lines represent 75^th^ percentile. *P = 0.03; ** P = 0.0002; *** P = 0.02. The associations are age and time of residence in malaria-endemic area (TRMAE) between individuals with high antibody levels (> 75th percentile) and low antibody levels (< 75th percentile). Reactivity indices were compared by Kruskal–Wallis followed by Mann–Whitney pairwise tests and correlations were represented by Spearman rank coefficients

In order to evaluate the associations of antibody response to GMZ2.6c, GLURP, MSP-3, and Pfs48/45 to personal, clinical, and epidemiological data, individuals from exposed (CZS, ML, and GJ) groups were all considered in a single group. Individuals with higher levels (> 75th percentile) of IgG and IgG3 antibodies against GMZ2.6c were older and presented a higher time of residence in malaria-endemic areas than individuals with lower IgG and IgG3 antibody levels (< 75th percentile) (mean ± standard deviation: 40 ± 18 versus 33 ± 15, P = 0.03, for IgG; 47 ± 17 versus 35 ± 16, P = 0.0002, for IgG3) (Fig. 11). IgG anti-GMZ2.6c antibody levels were negatively correlated (P = 0.01; r = − 0.557) to *P. falciparum* parasitaemia and positively correlated (P = 0.02; r = 0.1349) with the number of previous malaria infections. Levels of anti-GMZ2.6c IgG3 antibodies were also negatively associated (P = 0.02; r = − 0.3007) to parasitaemia. Individuals with higher levels (> 75th percentile) of anti-GLURP IgG3 antibodies were also older and presented a higher time of residence in the malaria-endemic areas (mean ± standard deviation: 46 ± 15 versus 36 ± 16, P = 0.02) (Fig. 11), but did not show different levels of parasitaemia when compared to those of low IgG3 antibody levels. No association was observed between antibody response to MSP-3 and Pfs48/45 and age, gender, time of exposure, presence of symptoms, number of previous malaria episodes, time elapsed since the last malaria episode and current or last infecting plasmodial species.

## Discussion

Given the complexity of the life cycle and of the parasite-host interactions, as well as the extensive parasite genetic variability, and the mechanisms it uses to evade the host immune response, an ideal malaria vaccine should be directed against antigens expressed in different development stages of the *Plasmodium*. In this sense, the recombinant GMZ2.6c protein has shown to be a promising multi-stage anti-malarial vaccine candidate. In this work, the antibody response profiles to the recombinant *Plasmodium falciparum* GMZ2.6c vaccine candidate in individuals living in Brazilian malaria-endemic areas, with different levels of transmission, was evaluated. The humoral response against the GMZ2.6c protein and its fragments, GLURP, MSP-3, and Pfs48/45, was assessed in individuals living in three cities of the Brazilian Amazon: Cruzeiro do Sul and Mâncio Lima, in the state of Acre, and Guajará, in the state of Amazonas.

Individuals were not differently distributed by sex, age, origin and previous exposure to malaria in Cruzeiro do Sul (CZS), Mâncio Lima (ML), and Guajará (GJ), although individuals from ML have reported a higher number of previous infections, even though ML is a highly endemic city like CZS. Most of the individuals reported previous infections by both *P. falciparum* and *P. vivax*, but *P. vivax* was the most prevalent infecting plasmodial species, reflecting the current malaria scenario in Brazil, where *P. vivax* is responsible for 89% of infections [39].

Most individuals from CZS, ML, and GJ had anti-GMZ2.6c antibodies, regardless of the immunoglobulin class, indicating that GMZ2.6c is widely recognized for naturally acquired antibodies from resident individuals. The antigenicity of GMZ2.6c seems to be independent of the endemicity levels, since the prevalence of individuals with antibodies that recognized GMZ2.6c was similar in the three study sites, with different endemicity levels.

Sixty-eight individuals (21 in CZS, 23 in ML, and 24 in GJ) had no antibodies that recognized GMZ2.6c (nor its individual fragments). The non-detection of anti-GMZ2.6c antibodies in these individuals could be associated to the absence of a previous contact with the parasite. However, of these, only 4 reported no previous malaria episodes and 10 reported only previous infection by *P. vivax*. The other individuals reported at least one *P. falciparum* infection. The non-detection of these antibodies could also be related to functional exhaustion among T and B cell subsets, well-described in viral infections [40–43] and equally considered for malaria [44–49]. Besides atypical memory B cells and the exhausted CD4 T cells, the absence of specific antibodies may depend on other factors, such as *P. falciparum* genetic polymorphisms, intensity of infection, coinfections, malnutrition, or human host genetic factors.

Clinical trials in naive subjects and in individuals naturally exposed to malaria in Africa to evaluate the safety and immunogenicity of GMZ2 showed a more potent response against GMZ2 when compared to its individual components [27, 28]. In the present study, a similar observation was made, where there was a higher prevalence of individuals with antibodies and higher levels of anti-GMZ2.6c antibodies, by comparing to its individual components, which may suggest an additive effect of GLURP, MSP-3, and Pfs48/45 when inserted in a same construct, and that GMZ2.6c may be a more promising anti-malarial vaccine candidate than its individual components. The data obtained in the present study corroborate a previous report of higher prevalence of individuals with antibodies anti-GMZ2 antibodies when compared to those against GLURP and MSP-3, in individuals naturally exposed to malaria in Ethiopia [32]. However, the possibility that the data observed in the present study are a simple reflex of the sum of the antibody response directed towards each of the antigens that make up GMZ2.6c, sufficient for individuals with antibody levels below the detection limit to exceed the threshold, cannot be excluded. Clarifying this point and evaluating the possibility of a synergistic effect of anti-GLURP, and anti-MSP-3 antibodies are ongoing in the laboratory.

Independent studies have evaluated the potential of humoral immune response against GLURP and MSP-3 in conferring clinical immunity to malaria in African endemic areas with different transmission intensities. A wide variety of results regarding the natural immunogenicity of GLURP and MSP-3 was observed. Some studies have reported the higher immunogenicity of MSP-3 when compared to GLURP-R0 [50, 51], while GLURP was more immunogenic in other studies [25, 52, 53]. However, in all of them, both MSP-3 and GLURP have been considered as endowed with greater immunogenicity properties. In the present study, both GLURP and MSP-3 have been shown to be widely recognized by antibodies, mainly in high-endemicity areas of CZS and ML.

In the present study, the prevalence of individuals with anti-Pfs48/45 antibodies ranged from 24 to 40%. These prevalences are lower than those (74.7%) observed in the Central Region of Ghana for the same Pfs48/45 construct (Pfs48/45.6c) (37), possibly reflecting differences in exposure to gametocytes as previously reported in a longitudinal study in a hypo-endemic area in Tanzania [54].

In this work, many individuals from the CZS, ML and GJ groups had IgG and IgM antibodies recognizing GMZ2.6c, contrasting with an uncommon IgA and IgE specific response. The data presented here also showed that anti-GMZ2.6c IgG antibodies were more frequent and present higher levels in the CZS group when compared to the ML and GJ groups. It is widely accepted that IgG antibodies are the main mediators of protection against clinical malaria [10, 55], and its important presence mainly in the CZS group may be reflecting a higher degree of anti-parasitic immunity. The higher prevalence of individuals with antibodies and higher antibody levels in the CZS group may be due to the genetic background of the study population since allelic forms of the Major Histocompatibility Complex (HLA) antigens may influence the host's ability to mount a naturally acquired humoral immune response [23, 56–58]. Additionally, the genetic variability of *P. falciparum* isolates circulating at variable levels in the Brazilian Amazonian Region may explain this difference in immune response in the CZS group, since genetic polymorphisms in B epitopes may influence the immunological properties of the antigen [59]. This remains to be elucidated.

The role of IgE antibodies in the outcome of malaria infection remains controversial and poorly understood. The levels of *P. falciparum*-specific IgE are proposed to be associated with protection as well as to participate in the pathogenesis of malaria [60–63]. Thus, the IgE antibody response against GMZ2.6c, GLURP, MSP-3 and Pfs48/45 was also assessed. The prevalence of individuals with anti-GMZ2.6c IgE antibodies and the levels of these antibodies were low (2.3 to 8.9%) in the three studied groups. However, a relatively high prevalence of individuals with IgE antibodies against GLURP and Pfs48/45 was verified, mainly in CZS. Some studies have reported that individual anti-*Anopheles* salivary gland IgE level is positively correlated with the antiplasmodial IgE antibody levels, and a hypothesis proposes that, during *Anopheles* bite, the injection of saliva containing pharmacologically active proteins and peptides predisposes individuals to the development of an antiplasmodial IgE response [64, 65].

It has been proposed that cytophilic IgG1 and IgG3 isotypes are more correlated with protective responses than the IgG2 and IgG4 (non-cytophilic) antibodies [10, 55]. Here, the profile of the IgG subclass against the GMZ2.6c, GLURP, MSP-3 and Pfs48/45 proteins was evaluated in all samples presenting IgG antibodies to the studied proteins. Naturally malaria-exposed individuals predominantly had anti-GMZ2.6c IgG1 and IgG3 cytophilic antibodies, an important fact considering that it has been proposed that not only the acquisition of anti-malaria protective immunity would result from a delicate balance between cytophilic/non cytophilic antibodies, but also that IgG2 and IgG4 non-cytophilic antibodies with the same specificity could block the effector mechanisms of the cytophilic ones [18, 66, 67]. Indeed, the interaction between cytophilic antibodies and Fc receptors on phagocytic cells can lead to cellular activation and to triggering of effector functions such as phagocytosis, cytokine and chemokine production, cytotoxicity, and the generation of reactive oxygen and nitrogen species [68]. Cytophilic antibodies may also mediate parasite death in cooperation with mononuclear cells through the antibody-dependent cellular inhibition mechanism (ADCI) besides the opsonization of *P. falciparum* merozoites [10, 69, 70].

Studies have shown that the inherent characteristics of plasmodial antigens can influence the molecular events that lead to the preferential induction of different IgG subclasses. The presence of both repetitive and polymorphic amino acid sequences predominantly induces an IgG3 response, whereas the absence of such polymorphic repeats predominantly induces IgG1 responses. Polymorphic antigens with no repetitive sequence induce an IgG1 + IgG3 response, with the predominance of IgG3, whereas conserved repetitive antigens tend to induce IgG1 or IgG1 + IgG3 responses [71, 72]. The data here obtained showed a predominantly cytophilic antibody response (IgG1 + IgG3) to GLURP, whereas MSP-3 induced a predominantly IgG1 response. These results may be due to the conserved nonrepetitive characteristic of GLURP contrasting with the repetitive nature and relatively more polymorphic of MSP-3 [73–75]. However, factors such as cumulative malaria exposure, age of individuals, HLA and the polymorphism of the FCγ receptor have also been shown to influence the distribution of IgG subclass against plasmodial antigens [71, 76]. In addition, specific combinations of cytokines and B-cell activators have shown to induce the switch to certain classes and subclass in experimental models [77]. In vitro experiments with human and murine B cells have identified cytokines that selectively induce the synthesis of a particular immunoglobulin class by the stimulation of DNA rearrangement and selective transcription of CH genes [78–81].

IgG and IgM antibodies levels against GMZ2.6c and GLURP were higher in parasitized individuals when compared to non-parasitized subjects. This result may reflect the stimulation of the immune system leading to the production of antiplasmodial antibodies, including anti-GLURP antibodies, due to infection. This booster, in response to infection, seems to induce a predominantly IgG1 and IgG3 antibodies that recognize GMZ2.6c and GLURP.

Classically, levels of antibodies against several plasmodial antigens seem to be associated with age and time of exposure to malaria in endemic regions [23, 82–88]. In the present study, individuals with higher levels of IgG and IgG3 anti-GMZ2.6c (> 75th percentile) antibodies were older and presented a longer time of residence in malaria-endemic areas than individuals with levels of antibody below the 75th percentile. Negative correlations were observed between parasitaemia and levels of IgG and IgG3, and a positive one was registered between the number of previous malaria infections and IgG antibodies against GMZ2.6c. Taken together, these data indicate that the prevalence of individuals with antibodies that recognize GMZ2.6c as well as the anti-GMZ2.6c antibody levels increase with exposure to infection and that these antibodies may contribute to parasite immunity. Considering the cross-sectional design of this work, additional studies are needed to confirm these findings. Moreover, the evaluation of the functional role of antibodies against MSP-3 and GLURP in inhibiting the in vitro growth of *P. falciparum* and of the anti-Pfs48/45 antibodies in inhibiting fertilization has been currently conducted in the laboratory and could also help to clarify this issue.

The analysis of the antibody response against the individual (GLURP, MSP-3 and Pfs48/45) proteins constituting the GMZ2.6c vaccine candidate did not provide evidence of a possible protective role of these antibodies, since no relationship was verified between prevalence or levels of specific antibodies and the presence or absence of parasites in the blood, the presence or absence of symptoms at the time of collection, parasitaemia, number of previous malaria episodes, time elapsed since the last malaria episode, current or last infecting plasmodial species. Associations between high anti-GLURP and anti-MSP3 antibody levels and low parasite densities in malaria patients living in the Central Region of Ghana have however been reported [33].

Taken together, the data presented here showed that GMZ2.6c protein is widely recognized by naturally acquired antibodies from individuals living in endemic areas of Brazil with different levels of transmission. The higher prevalence of individuals with antibodies that recognize GMZ2.6c and the higher levels of these antibodies when compared to their individual components may suggest an additive effect of GLURP, MSP-3, and Pfs48/45 when inserted in a same construct. Also, anti-GMZ2.6c antibodies seem to increase with exposure to malaria infection and may contribute to parasite immunity. These data highlight the importance of GMZ2.6c as a candidate for an anti-malarial vaccine. Additional studies in other endemic areas, with populations with different genetic backgrounds, besides the evaluation of the functional role of anti-GMZ2.6c antibodies, are important to confirm the potential of GMZ2.6c as an anti-malarial vaccine.

## Supplementary Information

**Additional file 1: Table S1.** Antigens and Enzyme-Linked Immunosorbent Assay antibody assays.

## Data Availability

The datasets supporting the conclusions of this article are included within the article and its additional files.

## References

[1] WHO. World Malaria Report. Geneva: World Health Organization; 2019. https://www.who.int/publications-detail/world-malaria-report-2019. Accessed 2 Jan 2020.

[2] Noedl H, Se Y, Schaecher K, Smith BL, Socheat D, Fukuda MM (2008). Evidence of artemisinin-resistant malaria in western Cambodia. N Engl J Med.

[3] Leang R, Taylor WR, Bouth DM, Song L, Tarning J, Char MC (2015). Evidence of *Plasmodium falciparum* malaria multidrug resistance to artemisinin and piperaquine in Western Cambodia: dihydroartemisinin-piperaquine open-label multicenter clinical assessment. Antimicrob Agents Chemother.

[4] Dondorp AM, Nosten F, Yi P, Das D, Phyo AP, Tarning J (2009). Artemisinin resistance in *Plasmodium falciparum* malaria. N Engl J Med.

[5] Dondorp AM, Yeung S, White L, Nguon C, Day NP, Socheat D (2010). Artemisinin resistance: current status and scenarios for containment. Nat Rev Microbiol.

[6] Vreden SG, Jitan JK, Bansie RD, Adhin MR (2013). Evidence of an incidence of day 3 parasitemia in Suriname: an indicator of the emerging resistance of *Plasmodium falciparum* to artemether. Mem Inst Oswaldo Cruz.

[7] Mathieu LC, Cox H, Early AM, Mok S, Lazrek Y, Paquet JC (2020). Local emergence in Amazonia of *Plasmodium falciparum* k13 C580Y mutants associated with *in vitro* artemisinin resistance. Elife.

[8] Matuschewski K (2017). Vaccines against malaria – still a long way to go. FEBS J.

[9] Baldwin SL, Roeffen W, Singh SK, Tiendrebeogo RW, Christiansen M, Beebe E (2016). Synthetic TLR4 agonists enhance functional antibodies and CD4+ T-cell responses against the Plasmodium falciparum GMZ2.6C multi-stage vaccine antigen. Vaccine.

[10] Theisen M, Adu B, Mordmüller B, Singh S (2017). The GMZ2 malaria vaccine: from concept to efficacy in humans. Expert Rev Vaccines.

[11] Quintana MDP, Chng J, Zandian A, Imam M, Hultenby K, Theisen M (2018). SURGE complex of Plasmodium falciparum in the rhoptry-neck (SURFIN4.2-RON4-GLURP) contributes to merozoite invasion. PLoS ONE.

[12] Imam M, Singh S, Kaushik NK, Chauhan VS (2014). *Plasmodium falciparum* merozoite surface protein 3: oligomerization, self-assembly, and heme complex formation. J Biol Chem.

[13] Borre MB, Dziegiel M, Hogh B, Petersen E, Rieneck K, Riley E (1991). Primary structure and localization of a conserved immunogenic *Plasmodium falciparum* glutamate rich protein (GLURP) expressed in both the preerythrocytic and erythrocytic stages of the vertebrate life cycle. Mol Biochem Parasitol.

[14] McColl DJ, Anders RF (1997). Conservation of structural motifs and antigenic diversity in the *Plasmodium falciparum* merozoite surface protein-3 (MSP-3). Mol Biochem Parasitol.

[15] van Dijk MR, Janse CJ, Thompson J, Waters AP, Braks JA, Dodemont HJ (2001). A central role for P48/45 in malaria parasite male gamete fertility. Cell.

[16] Graves PM, Carters R, Burkot TR, Quakyl IA, Kumar N (1988). Antibodies to *Plasmodium falciparum* gamete surface antigens in Papua New Guinea sera. Parasite Immunol.

[17] Ong SL, Zhang KY, Eida SJ, Graves PM, Dow C, Looker M (1990). The primary antibody response of malaria patients to *Plasmodium falciparum* sexual stage antigens which are potential transmission blocking vaccine candidates. Parasite Immunol.

[18] Oeuvray BC, Bouharoun-tayoun H, Gras-masse H, Bottius E, Kaidoh T, Aikawa M (1994). Merozoite Surface Protein-3: a malaria protein inducing antibodies that promote *Plasmodium falciparum* killing by cooperation with blood monocytes. Blood.

[19] Roeffen W, Mulder B, Teelen K, Bolmer M, Eling W, Targett GA (1996). Association between anti-Pfs48/45 reactivity and P. falciparum transmission-blocking activity in sera from Cameroon. Parasite Immunol.

[20] Theisen M, Soe S, Oeuvray C, Thomas AW, Vuust J, Danielsen S (1998). The glutamate-rich protein (GLURP) of *Plasmodium falciparum* is a target for antibody-dependent monocyte-mediated inhibition of parasite growth *in vitro*. Infect Immun.

[21] Oeuvray C, Theisen M, Rogier C, Trape JF, Jepsen S, Druilhe P (2000). Cytophilic immunoglobulin responses to *Plasmodium falciparum* glutamate-rich protein are correlated with protection against clinical malaria in Dielmo. Senegal Infect Immun.

[22] Theisen M, Soe S, Jessing SG, Okkels LM, Danielsen S, Oeuvray C (2000). Identification of a major B-cell epitope of the *Plasmodium falciparum* glutamate-rich protein (GLURP), targeted by human antibodies mediating parasite killing. Vaccine.

[23] Pratt-Riccio LR, Lima-Junior JC, Carvalho LJ, Theisen M, Espíndola-Mendes EC, Santos F (2005). Antibody response profiles induced by *Plasmodium falciparum* glutamate-rich protein in naturally exposed individuals from a Brazilian area endemic for malaria. Am J Trop Med Hyg.

[24] Bousema JT, Drakeley CJ, Kihonda J, Hendriks JCM, Akim NIJ, Roeffen W (2007). A longitudinal study of immune responses to *Plasmodium falciparum* sexual stage antigens in Tanzanian adults. Parasite Immunol.

[25] Pratt-Riccio LR, Bianco-Junior C, Totino PRR, Perce-da-Silva DS, Silva LA, Riccio EKP (2011). Antibodies against the *Plasmodium falciparum* glutamate-rich protein from naturally exposed individuals living in a Brazilian malaria-endemic area can inhibit *in vitro* parasite growth. Mem Inst Oswaldo Cruz.

[26] Baumann A, Magris MM, Urbaez M-L, Vivas-Martinez S, Durán R, Nieves T (2012). Naturally acquired immune responses to malaria vaccine candidate antigens MSP3 and GLURP in Guahibo and Piaroa indigenous communities of the Venezuelan Amazon. Malar J.

[27] Esen M, Kremsner PG, Schleucher R, Gässler M, Imoukhuede EB, Imbault N (2009). Safety and immunogenicity of GMZ2 – a MSP3-GLURP fusion protein malaria vaccine candidate. Vaccine.

[28] Mordmüller B, Szywon K, Greutelaers B, Esen M, Mewono L, Treut C (2010). Safety and immunogenicity of the malaria vaccine candidate GMZ2 in malaria-exposed, adult individuals from Lambaréné. Gabon Vaccine.

[29] Bélard S, Issifou S, Hounkpatin AB, Schaumburg F, Ngoa UA, Esen M (2011). A randomized controlled phase Ib trial of the malaria vaccine candidate GMZ2 in African children. PLoS ONE.

[30] Jepsen MPG, Jogdand PS, Singh SK, Esen M, Christiansen M, Issifou S (2013). The malaria vaccine candidate GMZ2 elicits functional antibodies in individuals from malaria endemic and non-endemic areas. J Infect Dis.

[31] Sirima SB, Mordmuller B, Milligan P, Ngoa UA, Kironde F, Atuguba F (2016). A phase 2b randomized, controlled trial of the efficacy of the GMZ2 malaria vaccine in African children. Vaccine.

[32] Mamo H, Esen M, Ajua A, Theisen M, Mordmüller B, Petros B (2013). Humoral immune response to *Plasmodium falciparum* vaccine candidate GMZ2 and its components in populations naturally exposed to seasonal malaria in Ethiopia. Malar J.

[33] Amoah LE, Nuvor SV, Obboh EK, Acquah FK, Asare K, Singh SK (2017). Natural antibody responses to *Plasmodium falciparum* MSP3 and GLURP(R0) antigens are associated with low parasite densities in malaria patients living in the Central Region of Ghana. Parasit Vectors.

[34] Secretaria de Vigilância em Saúde, Ministério da Saúde. Boletim Epidemiológico. Malária. 2020. https://www.gov.br/saude/pt-br/media/pdf/2020/dezembro/03/boletim_especial_malaria_1dez20_final.pdf. Acessed 21 Jan 2021.

[35] Snounou G (1996). Detection and identification of the four malaria parasite species infecting humans by PCR amplification. Methods Mol Biol.

[36] Ministry of Health of Brazil. Guide to malaria treatment in Brazil. 2020. http://bvsms.saude.gov.br/bvs/publicacoes/guia_tratamento_malaria_brasil.pdf. Accessed 20 Nov 2020.

[37] Acquah FK, Obboh EK, Asare K, Boampong JN, Nuvor SV, Singh SK (2017). Antibody responses to two new *Lactococcus lactis*-produced recombinant Pfs48/45 and Pfs230 proteins increase with age in malaria patients living in the Central Region of Ghana. Malar J.

[38] Singh SK, Tiendrebeogo RW, Chourasia BK, Kana IH, Singh S, Theisen M (2018). *Lactococcus lactis* provides an efficient platform for production of disulfide-rich recombinant proteins from *Plasmodium falciparum*. Microb Cell Fact.

[39] Secretaria de Vigilância em Saúde. Resumo Epidemiológico por Local de Notificação. 2019. http://portalsaude.saude.gov.br. Accessed 28 Jan 2020].

[40] El-Far M, Halwani R, Said E, Trautmann L, Doroudchi M, Janbazian L (2008). T-cell Exhaustion in HIV infection. Curr HIV/AIDS Rep.

[41] Moir S, Fauci AS (2009). B cells in HIV infection and disease. Nat Rev Immunol.

[42] Yi JS, Cox MA, Zajac AJ (2010). T-cell exhaustion: characteristics, causes and conversion. Immunology.

[43] Wherry EJ (2011). T cell exhaustion. Nat Immunol.

[44] Weiss GE, Crompton PD, Li S, Walsh LA, Moir S, Traore B (2009). Atypical memory B cells are greatly expanded in individuals living in a malaria-endemic area. J Immunol.

[45] Weiss GE, Traore B, Kayentao K, Ongoiba A, Doumbo S, Doumtabe D (2010). The *Plasmodium falciparum*-specific human memory B cell compartment expands gradually with repeated malaria infections. PLoS Pathog.

[46] Weiss GE, Clark EH, Li S, Traore B, Kayentao K, Ongoiba A (2011). A positive correlation between atypical memory B cells and *Plasmodium falciparum* transmission intensity in cross-sectional studies in Peru and Mali. PLoS ONE.

[47] Illingworth J, Butler NS, Roetynck S, Mwacharo J, Pierce SK, Bejon P (2013). Chronic exposure to *Plasmodium falciparum* is associated with phenotypic evidence of B and T cell exhaustion. J Immunol.

[48] Shankar EM, Vignesh R, Dash AP (2018). Recent advances on T-cell exhaustion in malaria infection. Med Microbiol Immunol.

[49] Holla P, Ambegaonkar A, Sohn H, Pierce SK (2019). Exhaustion may not be in the human B cell vocabulary, at least not in malaria. Immunol Rev.

[50] Soe S, Theisen M, Roussilhon C, Khin-Saw A, Druilhe P (2004). Association between protection against clinical malaria and antibodies to merozoite surface antigens in an area of hyperendemicity in Myanmar: complementarity between responses to merozoite surface protein 3 and the 220-kilodalton glutamate-rich protein. Infect Immun.

[51] Lusingu JPA, Vestergaard LS, Alifrangis M, Mmbando BP, Theisen M, Kitua AY (2005). Cytophilic antibodies to *Plasmodium falciparum* glutamate rich protein are associated with malaria protection in an area of holoendemic transmission. Malar J.

[52] Nebie I, Diarra A, Ouedraogo A, Soulama I, Bougouma EC, Tiono AB (2008). Humoral responses to *Plasmodium falciparum* blood-stage antigens and association with incidence of clinical malaria in children living in an area of seasonal malaria transmission in Burkina Faso. West Africa Infect Immun.

[53] Courtin D, Oesterholt M, Huismans H, Kusi K, Milet J, Badaut C (2009). The quantity and quality of African children’s IgG responses to merozoite surface antigens reflect protection against *Plasmodium falciparum* malaria. PLoS ONE.

[54] Bousema T, Roeffen W, Meijerink H, Mwerinde H, Mwakalinga S, van Gemert GJ (2010). The dynamics of naturally acquired immune responses to *Plasmodium falciparum* sexual stage antigens Pfs230 & Pfs48/45 in a low endemic area in Tanzania. PLoS ONE.

[55] Dobaño C, Santano R, Vidal M, Jiménez A, Jairoce C, Ubillos I (2019). Differential patterns of IgG subclass responses to *Plasmodium falciparum* antigens in relation to malaria protection and RTS. S vaccination Front Immunol.

[56] Banic DM, Goldberg AC, Pratt-Riccio LR, Oliveira-Ferreira J, Santos F, Gras-Masse H (2002). Human leukocyte antigen class II control of the immune response to p126-derived amino terminal peptide from *Plasmodium falciparum*. Am J Trop Med Hyg.

[57] Lima-Junior J, Pratt-Riccio LR (2016). Major histocompatibility complex and malaria: focus on *Plasmodium vivax* infection. Front Immunol.

[58] Pratt-Riccio LR, Perce-Da-Silva DS, Lima-Junior JC, Riccio EKP, Ribeiro-Alves M, Santos F (2017). Synthetic antigens derived from *Plasmodium falciparum* sporozoite, liver, and blood stages: naturally acquired immune response and human leukocyte antigen associations in individuals living in a Brazilian endemic area. Am J Trop Med Hyg.

[59] Pratt-Riccio LR, Perce-da-Silva DS, Lima-Junior JC, Theisen M, Santos F, Daniel-Ribeiro CT (2013). Genetic polymorphisms in the glutamate-rich protein of *Plasmodium falciparum* field isolates from a malaria-endemic area of Brazil. Mem Inst Oswaldo Cruz.

[60] Elghazali G, Perlmann H, Rutta AS, Perlmann P, Troye-Blomberg M (1997). Elevated plasma levels of IgE in *Plasmodium falciparum*-primed individuals reflect an increased ratio of IL-4 to interferon-gamma (IFNgamma)-producing cells. Clin Exp Immunol.

[61] Perlmann P, Perlmann H, Flyg BW, Hagstedt M, Elghazali G, Worku S (1997). Immunoglobulin E, a pathogenic factor in *Plasmodium falciparum* malaria. Infect Immun.

[62] Perlmann P, Perlmann H, ElGhazali G, Blomberg MT (1999). IgE and tumor necrosis factor in malaria infection. Immunol Lett.

[63] Bereczky S, Montgomery SM, Troye-Blomberg M, Rooth I, Shaw MA, Färnert A (2004). Elevated anti-malarial IgE in asymptomatic individuals is associated with reduced risk for subsequent clinical malaria. Int J Parasitol.

[64] Zeidner NS, Higgs S, Happ CM, Beaty BJ, Miller BR (1999). Mosquito feeding modulates Th1 and Th2 cytokines in flavivirus susceptible mice: an effect mimicked by injection of sialokinins, but not demonstrated in flavivirus resistant mice. Parasite Immunol.

[65] Lawaly R, Konate L, Marrama L, Dia I, Diallo D, Sarr FD (2012). Impact of mosquito bites on asexual parasite density and gametocyte prevalence in asymptomatic chronic *Plasmodium falciparum* infections and correlation with IgE and IgG titers. Infect Immun.

[66] Bouharoun-Tayoun H, Druilhe P (1992). Antibodies in *falciparum* malaria: what matters most, quantity or quality?. Mem Inst Oswaldo Cruz.

[67] Metzger WG, Okenu DMN, Cavanagh DR, Robinson JV, Bojang KA, Weiss HA (2003). Serum IgG3 to the *Plasmodium falciparum* merozoite surface protein 2 is strongly associated with a reduced prospective risk of malaria. Parasit Immunol.

[68] Ortega E, Soto-Cruz I (2007). Early biochemical events in leukocyte activation through receptors for IgG. Signal Transduct.

[69] Druilhe P, Pérignon JL (1994). Mechanisms of defense against P. falciparum asexual blood stages in humans. Immunol Lett.

[70] Bouharoun-Tayoun H, Oeuvray C, Lunel F, Druilhe P (1995). Mechanisms underlying the monocyte-mediated antibody-dependent killing of *Plasmodium falciparum* asexual blood stages. J Exp Med.

[71] Scopel KKG, Fontes CJF, Ferreira MU, Braga EM (2006). Factors associated with immunoglobulin G subclass polarization in naturally acquired antibodies to *Plasmodium falciparum* merozoite surface proteins: a cross-sectional survey in Brazilian Amazonia. Clin Vaccine Immunol.

[72] Tongren JE, Drakeley CJ, McDonald SLR, Reyburn HG, Manjurano A, Nkya WMM (2006). Target antigen, age, and duration of antigen exposure independently regulate immunoglobulin G subclass switching in malaria. Infect Immun.

[73] McColl DJ, Silva A, Foley M, Kun JF, Favaloro JM, Thompson JK (1994). Molecular variation in a novel polymorphic antigen associated with *Plasmodium falciparum* merozoites. Mol Biochem Parasitol.

[74] Huber W, Felger I, Matile H, Joachim Lipps H, Steiger S, Beck H-P (1997). Limited sequence polymorphism in the *Plasmodium falciparum* merozoite surface protein 31. Mol Biochem Parasitol.

[75] Pattaradilokrat S, Sawaswong V, Simpalipan P, Kaewthamasorn M, Siripoon N, Harnyuttanakorn P (2016). Genetic diversity of the merozoite surface protein-3 gene in *Plasmodium falciparum* populations in Thailand. Malar J.

[76] Aucan C, Traoré Y, Fumoux F, Rihet P (2001). Familial correlation of immunoglobulin G subclass responses to *Plasmodium falciparum* antigens in Burkina Faso. Infect Immun.

[77] Coffman RL, Lebman DA, Rothman P (1993). Mechansim and regulation of immunoglobulin isotype switching. Adv Immunol.

[78] Isakson BYPC, Vitetta ES (1982). T cell-derived B cell differentiation factor(s) Effect on the isotype switch of murine B cells. J Exp Med.

[79] Snapper CM, Finkelman FD (1990). Rapid loss of IgM expression by normal murine B cells undergoing IgG1 and IgE class switching after *in vivo* immunization. J Immunol.

[80] Stavnezer J (1996). Immunoglobulin class switching. Curr Opin Immunol.

[81] Honjo T, Kinoshita K, Muramatsu M (2002). Molecuar mechanism of class switch recombination: linkage with somatic hypermutation. Annu Rev Immunol.

[82] Egan AF, Morris J, Barnish G, Allen S, Greenwood BM, Kaslow DC (1996). Clinical immunity to *Plasmodium falciparum* malaria is associated with serum antibodies to the 19-kDa C-terminal fragment of the merozoite surface antigen, PfMSP-1. J Infect Dis.

[83] Branch OH, Udhayakumar V, Hightower AW, Oloo AJ, Hawley WA, Nahlen BL (1998). A longitudinal investigation of IgG and IgM antibody responses to the merozoite surface protein-1 19-kiloDalton domain of *Plasmodium falciparum* in pregnant women and infants: associations with febrile illness, parasitemia, and anemia. Am J Trop Med Hyg.

[84] Banic DM, Oliveira-Ferreira J, Pratt-Riccio LR, Conseil V, Gonçalves D, Fialho RR (1998). Immune response and lack of immune response to *Plasmodium falciparum* P126 antigen and its aminoterminal repeat in malaria-infected humans. Am J Trop Med Hyg.

[85] Roussilhon C, Oeuvray C, Muller-Graf C, Tall A, Rogier C, Trape JF (2007). Longterm clinical protection from *falciparum* malaria is strongly associated with IgG3 antibodies to merozoite surface protein 3. PLoS Med.

[86] John CC, Tande AJ, Moormann AM, Sumba PO, Lanar DE, Min XM (2008). Antibodies to pre-erythrocytic *Plasmodium falciparum* antigens and risk of clinical malaria in Kenyan children. J Infect Dis.

[87] Nebie I, Tiono AB, Diallo DA, Samandoulougou S, Diarra A, Konate AT (2008). Do antibody responses to malaria vaccine candidates influenced by the level of malaria transmission protect from malaria?. Trop Med Int Health.

[88] Osier FH, Fegan G, Polley SD, Murungi L, Verra F, Tetteh KKA (2008). Breadth and magnitude of antibody responses to multiple *Plasmodium falciparum* merozoite antigens are associated with protection from clinical malaria. Infect Immun.

