# Supplementary material for: Naturally acquired antibody response to a Plasmodium falciparum chimeric vaccine candidate GMZ2.6c and its components (MSP-3, GLURP, and Pfs48/45) in individuals living in Brazilian malaria-endemic areas
Source: Malar J. 2022 Jan 4;21:6. doi: 10.1186/s12936-021-04020-6 (PMC8729018; doi:10.1186/s12936-021-04020-6)
Supplement: Supplementary file 1 — Additional file 1: Table S1. Antigens and Enzyme-Linked Immunosorbent Assay antibody assays. [file 12936_2021_4020_MOESM1_ESM.pdf]

Supplementary Table 1: Antigens and Enzyme-Linked Immunosorbent Assay antibody assays

Antigen	*Coating*	*Washing*	*Blocking*	*Sera*	*Secondary antibody*
**GMZ2.6c**	1 µg/mL in PBS	4 times with PBS-T20 0,1%	1 hr with 3% (wt/vol) powdered-milk-containing PBS-T20 0,1% at RT	2 hr diluted (1:100) in 1% (wt/vol) powdered-milk-containing PBS-T20 0,1% at RT	1 hr diluted (1:1000) in 1% (wt/vol) powdered-milk-containing PBS-T20 0,1% at RT
**GLURP-R0**	1 µg/mL in carbonate-bicarbonate	4 times with PBS T20 0,05%	2 hr with 2% (wt/vol) powdered-milk-containing PBS at RT	1 hr diluted (1:100) in 1% (wt/vol) powdered-milk-containing PBS-T20 0,05% at RT	1 hr diluted (1:1000) in 1% (wt/vol) powdered-milk-containing PBS-T20 0,05% at RT
**MSP-3Ct**	5 µg/mL in carbonato-bicarbonato	3 times with PBS T20 0,05%	1 hr with 5% (wt/vol) powdered-milk-containing PBS-T20 0,05% at 37 ºC	1 hr diluted (1:100) in 1% (wt/vol) powdered-milk-containing PBS-T20 0,05% at 37ºC	1 hr diluted (1:1000) in 1% (wt/vol) powdered-milk-containing PBS-T20 0,05% at 37ºC
**Pfs48/45-6c**	1 µg/mL in PBS	4 times with PBS	1 hr with 5% (wt/vol) powdered-milk-containing PBS at RT	4 hr diluted (1:100) in 1% (wt/vol) powdered-milk-containing PBS-T20 0,1% at RT	1 hr diluted (1:1000) in 1% (wt/vol) powdered-milk-containing PBS at RT

PBS: Phosphate-buffered saline; T20: Tween 20; wt/vol: weight/volume; RT: Room temperature.
